# Interaction of Wnt Signaling with BMP/Smad Signaling during the Transition from Cell Proliferation to Myogenic Differentiation in Mouse Myoblast-Derived Cells

**DOI:** 10.1155/2013/616294

**Published:** 2013-06-20

**Authors:** Kumiko Terada, Satomi Misao, Naoki Katase, Shin-ichiro Nishimatsu, Tsutomu Nohno

**Affiliations:** Department of Molecular and Developmental Biology, Kawasaki Medical School, Kurashiki, Okayama 701-0192, Japan

## Abstract

*Background.* Wnt signaling is involved in muscle formation through *β*-catenin-dependent or -independent pathways, but interactions with other signaling pathways including transforming growth factor *β*/Smad have not been precisely elucidated. *Results.* As Wnt4 stimulates myogenic differentiation by antagonizing myostatin (GDF8) activity, we examined the role of Wnt4 signaling during muscle differentiation in the C2C12 myoblast cell line. Among several extrinsic signaling molecules examined in a microarray analysis of C2C12 cells during the transition from cell proliferation to differentiation after mitogen deprivation, *bone morphogenetic protein 4 (BMP4)* expression was prominently increased. *Wnt4* overexpression had similar effects on *BMP4* expression. BMP4 was able to inhibit muscle differentiation when added to the culture medium. BMP4 and noggin had no effects on the cellular localization of *β*-catenin induced by Wnt3a; however, the BMP4-induced phosphorylation of Smad1/5/8 was enhanced by Wnt4, but not by Wnt3a. The BMP antagonist noggin effectively stimulated muscle differentiation through binding to endogenous BMPs, and the effect of noggin was enhanced by the presence of Wnt3a and Wnt4. *Conclusion.* These results suggest that BMP/Smad pathways are modified through Wnt signaling during the transition from progenitor cell proliferation to myogenic differentiation, although Wnt/*β*-catenin signaling is not modified with BMP/Smad signaling.

## 1. Background

The Wnt and transforming growth factor (TGF) β signaling pathways have gained attention as potential therapeutic targets for cancer treatment and research interests in regenerative medicine and stem cell biology, because these extrinsic signals are implicated in cell fate determination [1–4]. The signaling interaction between the Wnt family and the TGFβ superfamily during morphogenetic processes in most animal embryos has been debated by developmental biologists [2–5]. At an initial step of axial determination, bone morphogenetic proteins (BMPs), members of the transforming growth factor-β (TGF-β) superfamily, and Wnt family members have been recognized as key mediators involved in the formation of the dorsal/ventral axis and the anterior/posterior axis, in addition to the fibroblast growth factor family and the hedgehog family [5, 6]. BMPs are known to regulate the proliferation, differentiation, and apoptosis of various types of cells during embryonic development and postnatal turnover or regeneration. The formation of skeletal muscle from paraxial mesoderm is influenced by signals from the neural tube and the dorsal ectoderm, including Wnt4 and BMP4, and by the determination of myogenic cell fate by intrinsic factors including *MyoD* and *Myf5* [7, 8]. In adult skeletal muscle, satellite cells proliferate and differentiate during myotube turnover through signaling by Wnt family members [9, 10].

 Mesenchymal stem cells are known sources of osteogenic and myogenic progenitor cells. The Wnt and BMP signaling pathways play key roles in stem cell maintenance and adult tissue homeostasis and in the commitment of mesenchymal stem cells to osteogenic or myogenic progenitor cell fates [7, 8]. Myogenic cell fate is determined by intrinsic and extrinsic signals, and the role of Wnt4 in myostatin-regulated muscle formation has been debated because Wnt4 signaling is independent of β-catenin signaling [11–16]. Although the commitment of mesenchymal progenitor cells to myoblast fate is known to be modulated through TGFβ superfamily members including myostatin, the role of Wnt signaling was only evaluated in a preliminary fashion with respect to signaling interactions [7–9].

Mouse myoblast-derived C2C12 cells can differentiate into myotubes under reduced mitogen conditions with low serum concentration. We used microarray analysis to determine the expression profile of C2C12 cells during myoblast differentiation. *BMP4* expression is upregulated after *Wnt4* overexpression and mitogen deprivation in C2C12 myoblast cells. We examined possible interactions between Wnt4 and BMP4 with respect to myogenic differentiation and found that BMP4/Smad signaling was affected by Wnt signaling during differentiation of myogenic progenitor cells.

## 2. Results

### 2.1. Wnt4-Overexpressing Cell Lines from C2C12 and Transcriptome Analysis

**Figure 1:**
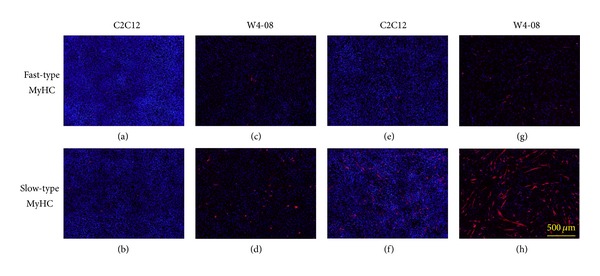
Myosin heavy chain (MyHC) expression in parental C2C12 cells and C2C12-derived cells permanently expressing Wnt4. Parental C2C12 cells (a, b, e, and f) and W4-08 cells (c, d, g, and h) were cultured for 2 days in proliferation medium containing 10% fetal bovine serum (a–d), or in differentiation medium containing 2% horse serum (e–h), and then immunohistochemically stained with anti-MyHC antibodies, followed by counterstaining with DAPI. Spontaneous expression of slow-type MyHC was evident in W4-08 cells in proliferation medium and intensified in differentiation medium, although the proliferation rates were greatly reduced compared to those of the parental C2C12 cells, as observed in the reduced number of nuclei (blue).

Mouse mesenchymal myogenic progenitor C2C12 cells can differentiate into myotubes after serum starvation under low-serum conditions. When a plasmid construct expressing *Wnt4* under control of the CMV promoter-enhancer was used to transfect C2C12 cells, we obtained several cell lines stably expressing *Wnt4* from the transfectants after several passages in the selection medium. Three cell lines named W4-02, W4-03, and W4-08, out of 14 candidate cell lines, were used for further studies. All of these cell lines showed a reduced rate of proliferation in rich medium, down to about half of the rate of the parental C2C12 cells. Although the expression level of Wnt4 protein was not high enough to be detected with anti-V5 antibodies, we detected *Wnt4* mRNA in these cell lines by reverse transcription PCR with Wnt4 primers (data not shown). When cultured in the proliferation medium and the differentiation medium containing 10% fetal bovine serum and 2% horse serum, respectively, these cells were able to differentiate spontaneously, as indicated by myosin heavy chain expression (MyHC) and a reduced number of cells (Figure 1). The parental C2C12 cells expressed MyHC very infrequently and proliferated more rapidly than the *Wnt4*-expressing cells.

**Figure 2:**
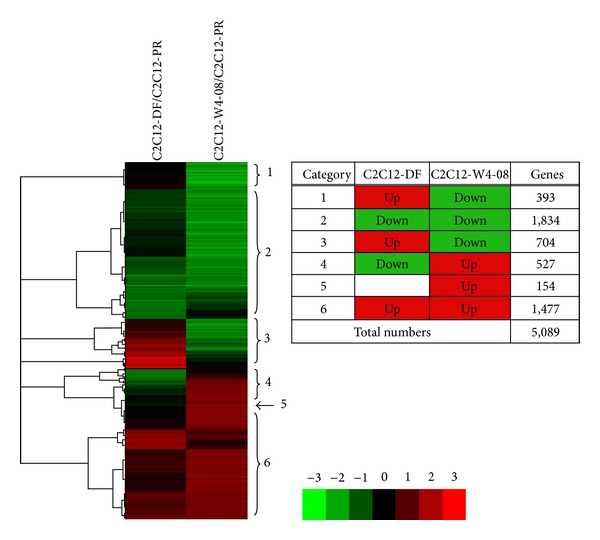
Summary of expression array analysis for C2C12 differentiation and Wnt4 expression. Red and green colors show upregulated and downregulated expression, respectively, with differentiation medium and Wnt4 overexpression. 1477 and 1836 genes were upregulated and downregulated, respectively, in differentiation medium and *Wnt4* overexpression at a twofold magnitude. Refer to original data in supplementary 1 for details.

When the total gene expression pattern was analyzed by hybridization with a mouse 24k cDNA microarray for transcriptome analysis, we found that the expression of the target genes involved in cell differentiation increased significantly during myoblast differentiation after the transition to the low-mitogen condition and *Wnt4* overexpression. Besides intrinsic signals related to myogenic differentiation, such as *MyoD1*, *myf5*, and *mrf4*, *BMP4* expression was elevated after *Wnt4* overexpression. Clustering analysis between C2C12 in proliferation medium (C2C12-PR) and C2C12 in differentiation medium (C2C12-DF) or W4-08 in proliferation medium (C2C12-W4-08) showed that thousands of genes were commonly upregulated or downregulated (Figure 2). The *myogenin* gene was upregulated in C2C12-DF, whereas the *Myf5* gene was upregulated in C2C12-W4-08, suggesting that serum starvation-induced myogenic differentiation and Wnt4-induced myogenic differentiation use overlapping but not identical signaling pathways. In *Wnt4*-expressing cells, 1477 differentiation-induced genes were also upregulated (Figure 2), including *Postn*, *Kif7*, *Kitl*, *Il2ra*, *Wnt4*, *Bmp4*, *Dcn*, *Cox6a2*, *Nov*, *Ogn*, and *Aspn* (See Supplementary 1 in the Supplementary Material available online at http://dx.doi.org/10.1155/2013/616294). On the other hand, 1834 differentiation-reduced genes were also downregulated in *Wnt4*-expressing cells (Figure 2), including *Mrpplf4*, *Jam4*, *Prkch*, *Nefl*, *Art3*, *Lrrn6c*, *Dlx3*, *Cd83*, *Ntsr2*, *Osbp2*, *Kpna4*, *E2f8*, and *Cck* (Supplementary 1). Among these upregulated genes, BMP4 is known to act as an extrinsic factor through autocrine or paracrine mechanisms to regulate cell differentiation through transmembrane receptors [17], and its elevated expression suggests the involvement of BMP signaling during myogenic differentiation. We therefore evaluated the effects of BMP4 protein on C2C12 differentiation.

### 2.2. Effects of BMP4 and Noggin on Myogenic Differentiation

**Figure 3:**
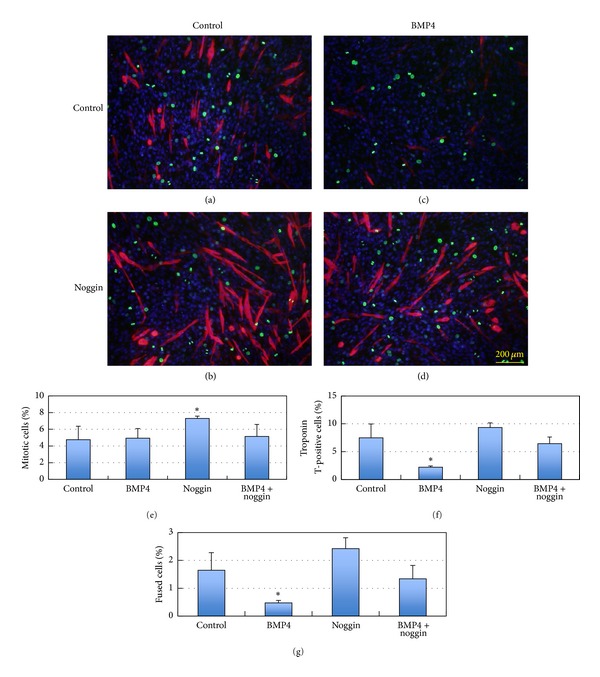
Effect of BMP4 and noggin on the myogenic differentiation of C2C12 cells. (a–d) The recombinant proteins were added to the proliferation medium at final concentrations of 5 ng/mL BMP4 and/or 50 ng/mL noggin and cultured in proliferation medium for 3 days, followed by immunostaining with anti-troponin T antibodies (red) and anti-phosphohistone H3 (Ser10) antibodies (green). BMP4 addition inhibited myogenic differentiation in proliferation medium. The number of cells expressing phosphorylated histone H3 was increased by adding noggin but not BMP4. (a) Control; (b) noggin; (c) BMP4; (d) noggin + BMP4. (e–g) The ratios of phosphohistone H3 (e) and troponin T-positive cells (f) to the total cell number were estimated. The number of multinuclear cells expressing troponin T (g) was also estimated to determine the ratio of fused cells among the total cells. Mean + SD (*n* = 3), *P < 0.05 versus control with Student's *t*-test.

**Figure 4:**
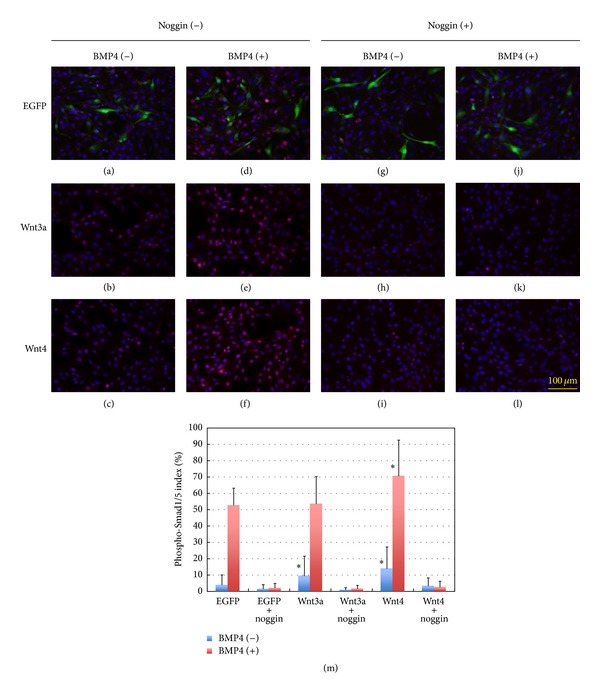
Effects of BMP4 and noggin on phospho-Smad1/5 expression with or without Wnt3a and Wnt4. (a–l) Recombinant adenoviruses were used to express *Wnt3a*, *Wnt4*, or *eGFP* (control) uniformly in C2C12 cells. Cells were infected 48 hours earlier with an MOI of 400, and the recombinant proteins were added as shown in Figure 3 with final concentrations of 5 ng/mL BMP4 and/or 250 ng/mL noggin. Two hours after BMP4 addition, immunostaining for phospho-Smad1/5 was carried out for counting. (a–c) Control; (d–f) BMP4; (g–i) noggin; (j–l) BMP4 + noggin. (m) The ratio of the nuclear phospho-Smad1/5-positive cells to the total cell number was estimated in three independent experiments counted for 6 fields each (mean + SD, *n* = 18, *P < 0.05 with Student's *t*-test).

When recombinant BMP4 protein was added to the culture medium, it had negative effects on the myogenic differentiation of progenitor cells, indicated by troponin T expression (Figure 3), as previously observed with myostatin, TGFβ, and BMP2 [18–20]. We used troponin T expression instead of MyHC expression hereafter because troponin T is expressed much earlier than MyHC in C2C12 cells even in the proliferation medium. The inhibitory effects of BMP4 addition to the culture medium on myogenic differentiation were blocked by noggin, and noggin itself promoted the differentiation of C2C12 cells (Figure 3). Smad1/5 phosphorylation induced by BMP4 was also inhibited by noggin, indicating an antagonistic activity of noggin at the level of BMP4 binding to the receptor complex (Figure 4). BMP4 showed negative effects on C2C12 differentiation, as indicated by the number of troponin T-expressing cells and of cells fusing to form multinuclear myotubes (Figure 3). The addition of noggin affected cell shape and promoted cell proliferation and differentiation, possibly by counteracting the effects of endogenous BMPs.

### 2.3. Wnt3a and Wnt4 Affect BMP4-Dependent Smad Signaling

**Figure 5:**
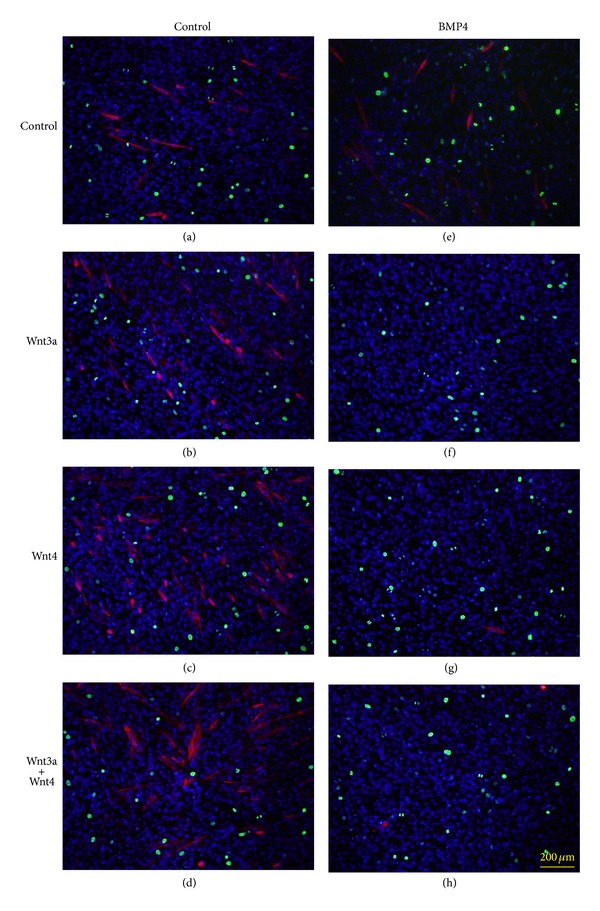
Effect of BMP4 on troponin T expression with or without Wnt3a and Wnt4. (a–h) The recombinant proteins were added as shown in Figure 3 with final concentrations of 125 ng/mL Wnt3a, 250 ng/mL Wnt4, and 5 ng/mL BMP4, alone or in combination as shown in the figure. Immunostaining and cell counting were carried out as described in Figure 3. Addition of BMP4 entirely abolished troponin T expression in the presence or absence of Wnt3a and/or Wnt4. (a) Control; (b) Wnt3a; (c) Wnt4; (d) Wnt3a + Wnt4; (e) BMP4; (f) Wnt3a + BMP4; (g) Wnt4 + BMP4; (h) Wnt3a + Wnt4 + BMP4.

**Figure 6:**
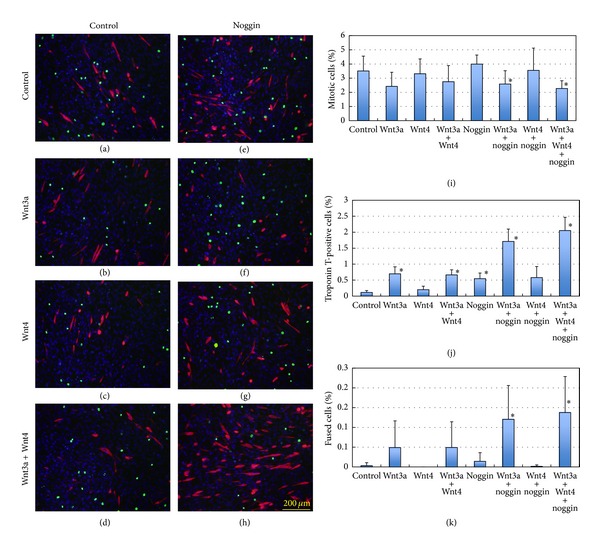
Effect of noggin on troponin T expression with or without Wnt3a and Wnt4. (a–h) The recombinant proteins were added as shown in Figure 3 with final concentrations of 125 ng/mL Wnt3a, 250 ng/mL Wnt4, and 50 ng/mL noggin, alone or in combination as shown in the figure. Immunohistochemical staining and cell counting were carried out as described in Figure 3. Addition of noggin elevated troponin T expression in the presence or absence of Wnt3a and/or Wnt4. (a) Control; (b) Wnt3a; (c) Wnt4; (d) Wnt3a + Wnt4; (e) noggin; (f) Wnt3a + noggin; (g) Wnt4 + noggin; (h) Wnt3a + Wnt4 + noggin. (i–k) The ratios of phosphohistone H3 (i) and troponin T-positive cells (j) to the total cell number were counted. Multinuclear cells expressing troponin T (k) were also estimated to determine the ratio of fused cells to the total cell number. Mean + SD (*n* = 6), *P < 0.01 versus control with Student's *t*-test.

The effects of BMP4 and noggin on phospho-Smad1/5 expression were examined in the presence or absence of Wnt3a and Wnt4. Wnt3a either synergizes with or opposes the effects of Wnt4 on myogenic differentiation, depending on the C2C12 cellular context (Figures 5 and 6). When BMP4 was added in combination with Wnt3a and/or Wnt4, C2C12 differentiation into troponin T-expressing cells in the proliferation medium was markedly inhibited, and the inhibitory effects were most evident in the presence of Wnt3a and Wnt4 (Figure 5). On the other hand, noggin stimulated myogenic differentiation, as indicated by enhanced expression of troponin T (Figure 6). The stimulatory effects on troponin T expression were most remarkable in the presence of Wnt3a and Wnt4 (Figure 6). Thus, the Wnt family members modulated myogenic differentiation through a positive influence on BMP4/Smad signaling (Figure 4). The effects of BMP4 on phospho-Smad1/5 expression were modified by Wnt4 but not by Wnt3a (Figure 4), although Wnt3a and Wnt4 both effectively stimulated basal and endogenous Smad1/5 signaling in C2C12 cells (Figure 4).

### 2.4. Wnt3a-Induced β-Catenin Localization Is Not Influenced by BMP4 and Noggin

**Figure 7:**
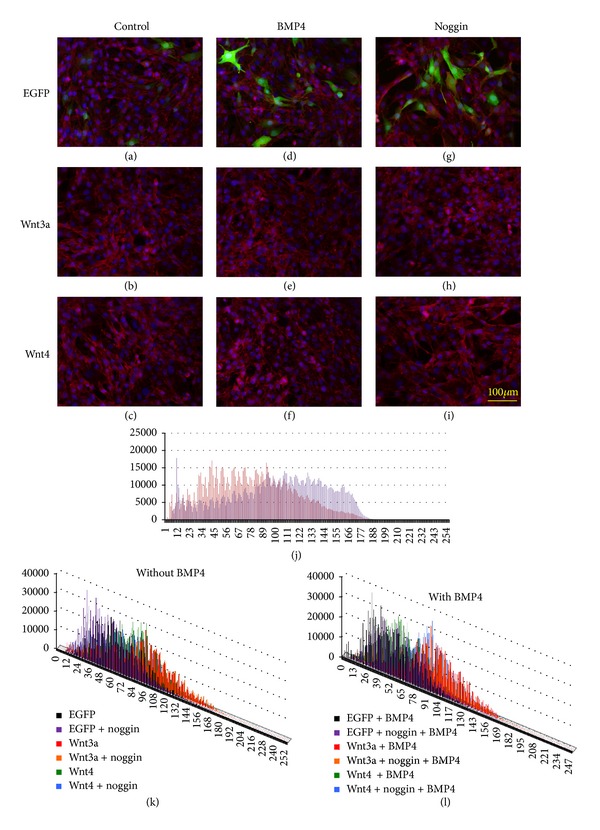
Effects of BMP4 and noggin on β-catenin localization with or without Wnt3a and Wnt4. (a–i) Recombinant adenoviruses were used to express *Wnt3a*, *Wnt4*, or *eGFP* (control) uniformly in C2C12 cells. Cells were infected 48 hours earlier with an MOI of 400, and the recombinant proteins were added as shown in Figure 3 with final concentrations of 5 ng/mL BMP4 and/or 250 ng/mL noggin. Two hours after BMP4 addition, immunostaining for β-catenin was carried out for counting. (j) Comparison of the β-catenin signals after adenovirus-mediated expression of *Wnt3a* and *eGFP*, after pixel intensity analysis for immunohistochemical signals with BZ-II Analyzer, shown in abscissa for signal intensity and ordinate for frequency. Wnt3a intensified signals to a higher level. (k and l) Comparison of the β-catenin signals after adenovirus-mediated expression of *Wnt3a*, *Wnt4,* and *eGFP*, in the presence or absence of BMP4 and/or noggin. Immunostaining for β-catenin was analyzed by digitizing the signals with BZ-II Analyzer for comparison. Typical results are shown. (k) Without BMP4; (l) with BMP4.

Here, we used recombinant adenoviruses expressing *Wnt3a* and *Wnt4* instead of the recombinant proteins, because the subcellular localization and detection of β-catenin were much easier with a low but uniform expression of *Wnt* genes in the target cells [16]. As shown in previous reports, *Wnt3a* overexpression resulted in elevated proliferation rates in myoblast cells while preventing myogenic differentiation accompanying nuclear localization of β-catenin, whereas *Wnt4* overexpression counteracted the effects of *Wnt3a*, resulting in reduced rates of proliferation and enhanced differentiation [16]. To observe the effects of BMP/Smad signaling on Wnt/β-catenin signaling, we used adenovirus-mediated expression of *Wnt3a* and *Wnt4*, since viral expression of the *Wnt* genes resulted in consistent elevation of β-catenin signaling (Figure 7). When *Wnt3a* was overexpressed in C2C12 cells, troponin T expression was significantly enhanced compared to control cells expressing *eGFP* instead of *Wnt* (Figure 6). On the contrary, *Wnt4* expression showed minor effect on myogenic differentiation, as indicated by the increased number of troponin T-expressing cells and of cells fusing to form multinuclear myotubes (Figure 6). When BMP4 and noggin were added to the culture medium after overexpression of *Wnt3a* and *Wnt4* with adenovirus vectors, β-catenin signals in C2C12 cells were not affected (Figure 7), indicating that Wnt/β-catenin signaling was not affected or modified by BMP/Smad signaling.

## 3. Discussion

We established *Wnt4*-expressing cells from C2C12 cells after transfection of an expression construct followed by clonal selection. These cells spontaneously differentiated to form myotubes, characterized by troponin T and myosin heavy chain expression, even in serum-rich medium. The growth rate of the *Wnt4*-expressing cells decreased to about half of that of the original cells in proliferation medium, because the *Wnt4*-overexpressing cells had a tendency to spontaneously differentiate. Thus, the *Wnt4*-overexpressing cells had the unique phenotype of expressing myosin heavy chain even in the mitogen-containing proliferation medium, in contrast to the previously observed phenotype of C2C12 cells overexpressing *Wnt3a* [21]. *Wnt4* overexpression appears to override or dominate the regulation of the C2C12 phenotypes in the proliferation and differentiation states.

*Wnt3a* overexpression is known to counteract the BMP2-induced inhibition of myotube formation in C2C12 cells switched to low-serum medium, and BMP2-induced* Id1* expression was decreased in *Wnt3a*-expressing cells but not in *Wnt5a*-expressing cells [21]. BMP2 and BMP4 have identical activity in binding BMP receptors and activate Smad1/5/8 in C2C12 cells [22], although the induction of differentiation after *Wnt4* expression or mitogen deprivation elevated *BMP4* but not *BMP2* expression. Signaling interaction with Wnt family members resulted in different but overlapping consequences, depending on activation of the canonical or the noncanonical Wnt pathway [23–25].

Wnt4 was shown to counteract the effect of myostatin on myogenic differentiation either upstream or downstream of the Smad2/3 pathways [8, 11, 12, 15], whereas elevated levels of BMP2/4 resulted in Smad1/5/8 activation and regulated the transition of progenitor cells from a myogenic to an osteogenic cell fate [22, 24, 26]. Signal crosstalk between BMP/Smad and Wnt/β-catenin is partially implicated in the commitment of myogenic progenitor cells to the formation of myotubes; a functional interaction between the BMP and Wnt families is known in paraxial mesoderm differentiation and cell fate determination during early embryogenesis [3, 5]. Wnt3a-dependent subcellular localization of β-catenin plays a role in progenitor cell proliferation as one of the determinants of myogenic cell fate, independent of BMP and noggin [23, 27]. However, Wnt4 modified BMP4-dependent Smad1/5 phosphorylation, suggesting that noncanonical Wnt signaling plays a role in BMP2/4-dependent signaling during myogenic differentiation, and functional Notch signaling may be implicated in this process [28]. However, discrepancy on Smad1/5 phosphorylation after *Wnt3a* and *Wnt4* expression is not simply interpreted at present in terms of BMP4-induced Smad1/5 phosphorylation, suggesting indirect interaction between BMP/Smad signaling and Wnt signaling. Although calcium signaling is crucial in myoblast differentiation, there is no evidence in C2C12 cells whether noncanonical Wnt4 plays a role in calcium signaling as known for Wnt5a [1–3]. 

Noggin is a BMP-binding protein that antagonizes Smad1/5/8 phosphorylation at the transmembrane receptor complexes and can stimulate muscle differentiation through antagonism of endogenous BMPs. This effect of noggin was enhanced in the presence of Wnt3a and Wnt4. Since the Wnt/β-catenin pathway was not modified by BMP/Smad signaling during the transition from progenitor cell proliferation to myogenic differentiation, direct signal crosstalk is unlikely and probably independently regulated. BMP4/Smad signaling is presumed to counteract Wnt4-promoted myogenic differentiation. However, a positive correlation was found between noncanonical Wnt4 signaling and BMP4-induced Smad1/5 phosphorylation. Wnt4 was shown to counteract Wnt3a in myoblast differentiation [16], and Wnt4, but not Wnt3a, potentiated BMP4-dependent Smad1/5 phosphorylation, suggesting that noncanonical Wnt4 signaling is involved in myogenic differentiation in concert with Wnt3a/β-catenin.

## 4. Conclusions

 Wnt/β-catenin signaling is involved in various aspects of skeletal muscle development and regeneration [7–9]. The characterization of signaling interactions provides insights for the development of therapeutics for regenerative medicine based on the fundamental cascades during myogenic proliferation and differentiation. Our results suggest that noncanonical Wnt signaling interacts with BMP signaling through the regulation of Smad1/5 phosphorylation and acts in addition to Wnt/β-catenin signaling in myogenic turnover.

## 5. Methods

### 5.1. Cell Culture and Cloning of Wnt4-Expressing Transfectants

The C2C12 cell line (myoblast-like cell line from the C3H mouse) was purchased from the RIKEN Cell Bank (RIKEN, Wako, Japan) and cultured in Dulbecco's modified Eagle's medium (DMEM) supplemented with 10% fetal bovine serum (FBS, Nichirei Corp., Tokyo). After 12 to 24 h of subculture, cells were transfected with an expression vector bearing V5-tagged *Wnt4* cDNA (P_CMV_-Wnt4-V5-P_PGK_-blasticidin-SV40pA). Transfected C2C12 cells were cultured in DMEM containing 10% FBS and blasticidin (Life Technologies Co.). Fourteen clones were selected and about half of them were used for further analysis. *Wnt4*-expressing stable transfectants were obtained after serial passages in selection medium containing blasticidin for 4 to 5 weeks.

### 5.2. Expression Analysis

Transcriptome analysis was carried out using total RNA extracted from C2C12 cells under proliferation or differentiation conditions, and the results were compared between parental and *Wnt4*-expressing C2C12-derived cells. Cells were grown either in proliferation medium containing 10% FBS in DMEM or in differentiation medium containing 2% horse serum (Sigma-Aldrich) in DMEM and cultured for two days in 100 mm diameter dishes. Total RNA was extracted from cultured cells using Isogen (Nippon Gene Co., Tokyo, Japan) according to the manufacturer's instructions. Expression profiles were examined under the following conditions: C2C12 proliferating in rich medium; C2C12 differentiating in poor medium; *Wnt4*-overexpressing C2C12 cell line proliferating in rich medium. Labeling, hybridization, scanning, and data processing were carried out with Toray 3D Gene (Toray Industries, Inc., Tokyo). Minimum information about a microarray experiment-(MIAME-) compliant array data including raw data is deposited in the Gene Expression Omnibus (GEO) at NCBI with accession number GSE40456.

### 5.3. Recombinant Virus Preparation and Treatment

Adenoviruses carrying Wnt cDNAs were prepared using the ViraPower Adenovirus Expression System (Life Technologies Co.), and recombinant adenoviruses expressing *Wnt3a*, *Wnt4, *and *eGFP* cDNAs were constructed as described previously [16]. In brief, 293A cells were used to propagate recombinant adenovirus until the viral titer reached about 10^9^ pfu/mL. C2C12 cells were infected with a multiplicity of infection (MOI) of 400 because of poor infection efficiency in C2C12 cells. Recombinant Wnt3a, Wnt4, and noggin Fc chimera were obtained from R&D Systems through Cosmo Bio Co., Ltd. (Tokyo, Japan), recombinant BMP4 was obtained from HumanZyme (Chicago, IL, USA), and the recombinant proteins were used according to the manufacturers' recommendations. An adenovirus vector expressing eGFP was used as a negative control for virally mediated transgene expression, and green fluorescence was monitored to estimate transgene expression.

### 5.4. Immunofluorescence Detection

Cells were fixed in phosphate-buffered saline (PBS) containing 4% paraformaldehyde. After three washes with PBS, cells were immunohistochemically stained as previously described [16], using anti-fast myosin heavy chain (M4276, Sigma-Aldrich, Saint Louis, MO, USA), anti-slow myosin heavy chain (M8421, Sigma-Aldrich), anti-troponin T (MAB1487, clone TT-98, Abnova, Walnut, CA, USA), anti-β-catenin (C2206, Sigma-Aldrich), anti-phospho-Smad1/5, and anti-phospho- (Ser10) histone H3 (382159, Calbiochem, Millipore) antibodies at 1 : 100, 1 : 200, 1 : 40, 1 : 200, 1 : 1000, and 1 : 500 dilutions, respectively. After washing, cells were incubated with a 1 : 250 dilution of secondary Alexa Fluor 594- or 488-conjugated goat anti-mouse or anti-rabbit IgG antibodies (A11032, A11037, A11029, Molecular Probes, Invitrogen, Carlsbad, CA, USA). Cell nuclei were stained with 1 *μ*g/mL 4′, 6′-diamino-2-phenylindole solution (DAPI, Dojindo, Kumamoto, Japan). Fluorescent images were taken using an All-in-One Fluorescence Microscope BZ-9000 (Keyence, Osaka, Japan). Statistical significance of the immunostained cell number was evaluated with Student's *t*-test.

## Supplementary Material

Expression profiles after clustering analysis for mouse whole transcripts. Messenger RNAs from C2C12 in proliferating medium (C2C12-PR), C2C12 in differentiation medium (C1C12-DF), and W4-08 in proliferating medium (C2C12-W4-08) were analyzed with the 3D-GeneTM mouse oligo chip 24K (Toray Industries, Inc., Tokyo).
